# Supplementary material for: Genetic diversity of Cryptosporidium spp. in non-human primates in rural and urban areas of Ethiopia
Source: PLoS One. 2022 Apr 14;17(4):e0267103. doi: 10.1371/journal.pone.0267103 (PMC9009656; doi:10.1371/journal.pone.0267103)
Supplement: Attachment — Submitted filename: Response to Reviewers.docx [file pone.0267103.s001.docx]

PONE-D-21-37489

Genetic diversity of *Cryptosporidium spp.* in non-human primates in rural and urban areas of Ethiopia

**PLOS ONE**

Dear Dr. Benjamin M. Rosenthal

Thank you for your comments and sending us the reviewer's comments. We thank also the reviewer for the constructive comments. We have revised the manuscript following the reviewer's suggestions. We described these changes in the below paragraphs. We hope that you will find our responses are acceptable, and we are looking forward to hearing your decision.

**Editors**

1. Please ensure that your manuscript meets PLOS ONE's style requirements, including those for file naming. The PLOS ONE style templates can be found at

<https://journals.plos.org/plosone/s/file?id=wjVg/PLOSOne_formatting_sample_main_body.pdf> and

<https://journals.plos.org/plosone/s/file?id=ba62/PLOSOne_formatting_sample_title_authors_affiliations.pdf>

**Response**: We have checked the formatting of the manuscript. The revised manuscript meets the Plos one’s style requirements.

2. In your Methods section, please provide additional information regarding the permits you obtained for the work. Please ensure you have included the full name of the authority that approved the field site access and, if no permits were required, a brief statement explaining why.

**Response**: We acknowledge the editor’s comment and included the text below in the methods section.

“We performed non-invasive sampling [24] and collected only a fresh sample from the monkey's feces dripping off the floor. Verbal approval was obtained from the residents of Wurgissa and Hawassa, who live near the sampling site, and from local authorities (i.e., Hawassa city agriculture office and Wurgissa Kebele administration).” (See line 64-66 pages 5, 6)

3. Please include your tables as part of your main manuscript and remove the individual files. Please note that supplementary tables (should remain/ be uploaded) as separate "supporting information" files.

**Response**: We have included the table as part of the main manuscript in the revised submission.

4. Thank you for stating the following financial disclosure:

“NO”

At this time, please address the following queries:

a)        Please clarify the sources of funding (financial or material support) for your study. List the grants or organizations that supported your study, including funding received from your institution.

b)        State what role the funders took in the study. If the funders had no role in your study, please state: “The funders had no role in study design, data collection and analysis, decision to publish, or preparation of the manuscript.”

c)        If any authors received a salary from any of your funders, please state which authors and which funders.

d)        If you did not receive any funding for this study, please state: “The authors received no specific funding for this work.”

Please include your amended statements within your cover letter; we will change the online submission form on your behalf.

**Response**: This study didn’t receive funding support. So, we have included the text “The authors received no specific funding for this work” in the cover letter.

5. In your Data Availability statement, you have not specified where the minimal data set underlying the results described in your manuscript can be found. PLOS defines a study's minimal data set as the underlying data used to reach the conclusions drawn in the manuscript and any additional data required to replicate the reported study findings in their entirety. All PLOS journals require that the minimal data set be made fully available. For more information about our data policy, please see <http://journals.plos.org/plosone/s/data-availability>.

Upon re-submitting your revised manuscript, please upload your study’s minimal underlying data set as either Supporting Information files or to a stable, public repository and include the relevant URLs, DOIs, or accession numbers within your revised cover letter. For a list of acceptable repositories, please see <http://journals.plos.org/plosone/s/data-availability#loc-recommended-repositories>. Any potentially identifying patient information must be fully anonymized.

Important: If there are ethical or legal restrictions to sharing your data publicly, please explain these restrictions in detail. Please see our guidelines for more information on what we consider unacceptable restrictions to publicly sharing data: <http://journals.plos.org/plosone/s/data-availability#loc-unacceptable-data-access-restrictions>. Note that it is not acceptable for the authors to be the sole named individuals responsible for ensuring data access.

We will update your Data Availability statement to reflect the information you provide in your cover letter.

You may need to state your data availability statement here and mention that you will specify this in the additional information section when you submit the revised manuscript.

**Response**: we have included data availability statement which reads ‘All relevant data are within the manuscript.” in the cover letter. We have also included the same statement as additional information during the online submission

6. We note that Figure 1 in your submission contain map images which may be copyrighted. All PLOS content is published under the Creative Commons Attribution License (CC BY 4.0), which means that the manuscript, images, and Supporting Information files will be freely available online, and any third party is permitted to access, download, copy, distribute, and use these materials in any way, even commercially, with proper attribution. For these reasons, we cannot publish previously copyrighted maps or satellite images created using proprietary data, such as Google software (Google Maps, Street View, and Earth). For more information, see our copyright guidelines: <http://journals.plos.org/plosone/s/licenses-and-copyright>.

We require you to either (1) present written permission from the copyright holder to publish these figures specifically under the CC BY 4.0 license, or (2) remove the figures from your submission:

a. You may seek permission from the original copyright holder of Figure 1 to publish the content specifically under the CC BY 4.0 license.

We recommend that you contact the original copyright holder with the Content Permission Form (<http://journals.plos.org/plosone/s/file?id=7c09/content-permission-form.pdf>) and the following text:

“I request permission for the open-access journal PLOS ONE to publish XXX under the Creative Commons Attribution License (CCAL) CC BY 4.0 (<http://creativecommons.org/licenses/by/4.0/>). Please be aware that this license allows unrestricted use and distribution, even commercially, by third parties. Please reply and provide explicit written permission to publish XXX under a CC BY license and complete the attached form.”

Please upload the completed Content Permission Form or other proof of granted permissions as an "Other" file with your submission.

In the figure caption of the copyrighted figure, please include the following text: “Reprinted from [ref] under a CC BY license, with permission from [name of publisher], original copyright [original copyright year].”

b. If you are unable to obtain permission from the original copyright holder to publish these figures under the CC BY 4.0 license or if the copyright holder’s requirements are incompatible with the CC BY 4.0 license, please either i) remove the figure or ii) supply a replacement figure that complies with the CC BY 4.0 license. Please check copyright information on all replacement figures and update the figure caption with source information. If applicable, please specify in the figure caption text when a figure is similar but not identical to the original image and is therefore for illustrative purposes only.

The following resources for replacing copyrighted map figures may be helpful:

USGS National Map Viewer (public domain): <http://viewer.nationalmap.gov/viewer/>

The Gateway to Astronaut Photography of Earth (public domain): <http://eol.jsc.nasa.gov/sseop/clickmap/>

Maps at the CIA (public domain): <https://www.cia.gov/library/publications/the-world-factbook/index.html> and <https://www.cia.gov/library/publications/cia-maps-publications/index.html>

NASA Earth Observatory (public domain): <http://earthobservatory.nasa.gov/>

Landsat: <http://landsat.visibleearth.nasa.gov/>

USGS EROS (Earth Resources Observatory and Science (EROS) Center) (public domain): [http://eros.usgs.gov/#](http://eros.usgs.gov/)

Natural Earth (public domain): <http://www.naturalearthdata.com/>

**Response**: We acknowledge the editor’s suggestion and removed Fig 1 from the revised submission.

7. Please upload a new copy of Figure 2 as the detail is not clear. Please follow the link for more information: <https://blogs.plos.org/plos/2019/06/looking-good-tips-for-creating-your-plos-figures-graphics/>.

**Response**: We acknowledge the lack of clarity of the details in Fig 2, but we have removed the figure from the revised submission at the reviewer’s recommendation.

8. Please review your reference list to ensure that it is complete and correct. If you have cited papers that have been retracted, please include the rationale for doing so in the manuscript text, or remove these references and replace them with relevant current references. Any changes to the reference list should be mentioned in the rebuttal letter that accompanies your revised manuscript. If you need to cite a retracted article, indicate the article’s retracted status in the References list and also include a citation and full reference for the retraction notice.

**Response:** We have checked the references to meet to Plos one’s style requirement. There are no retracted references but have included one new reference to the list (reference # 24).

Reviewers' comments:

Comments to the Author

1. Is the manuscript technically sound, and do the data support the conclusions?

The manuscript must describe a technically sound piece of scientific research with data that supports the conclusions. Experiments must have been conducted rigorously, with appropriate controls, replication, and sample sizes. The conclusions must be drawn appropriately based on the data presented.

Reviewer #1: Partly

2. Has the statistical analysis been performed appropriately and rigorously?

Reviewer #1: Yes

3. Have the authors made all data underlying the findings in their manuscript fully available?

The [PLOS Data policy](http://www.plosone.org/static/policies.action#sharing) requires authors to make all data underlying the findings described in their manuscript fully available without restriction, with rare exception (please refer to the Data Availability Statement in the manuscript PDF file). The data should be provided as part of the manuscript or its supporting information, or deposited to a public repository. For example, in addition to summary statistics, the data points behind means, medians and variance measures should be available. If there are restrictions on publicly sharing data—e.g. participant privacy or use of data from a third party—those must be specified.

Reviewer #1: Yes

4. Is the manuscript presented in an intelligible fashion and written in standard English?

PLOS ONE does not copyedit accepted manuscripts, so the language in submitted articles must be clear, correct, and unambiguous. Any typographical or grammatical errors should be corrected at revision, so please note any specific errors here.

Reviewer #1: No

5. Review Comments to the Author

Please use the space provided to explain your answers to the questions above. You may also include additional comments for the author, including concerns about dual publication, research ethics, or publication ethics. (Please upload your review as an attachment if it exceeds 20,000 characters)

**Reviewer #1**

The manuscript “Genetic diversity of Cryptosporidium spp. in non-human primates in rural and urban areas of Ethiopia” describes the results of a molecular survey of Cryptosporidium from two species of NHPs. The study is straightforward, methods seems appropriate, and the data from this study are of interest and value. However, the manuscript needs improvement in how the results have been presented and described before it is suitable for publication. My specific comments are as follows:

1. Data are from 2 species of NHP, Chlorocebus aethiops (vervet monkey) and Colobus guereza (mantled guereza). Methods seems to only describe collection of samples for vervet monkey. Also there are far more samples from vervet monkeys (177) than mantled guerezas (8). But description of results combines data from both species. Overall, the manuscript would be improved by more clearly describing the data from each species included in the study.

**Response**: We have made clear that samples were collected from vervet monkey and mantled guereza in the method section and presented the data separately for the two species. We have copied below the added text in the methods and results section of the revised submission

Method:

“The study subjects were free-ranging wild vervet and colobus monkeys found in Hawassa town and the Vervet monkeys living in a rural village ‘Wurgissa’ when collecting the sample. Visitors, and people in the open recreation area of Hawassa town often feed the monkeys with leftover food. (see line 75-77; page 5 )

Results

“Out of 51 fecal samples tested positive for *Cryptosporidium* infection, 47 were obtained from *Chlorocebus aethiops* and 4 from *Colobus guereza*. ( see line 144-145 page 8)

“Subtyping analysis of the *C. parvum* and *C. hominis* isolates from *Chlorocebus aethiops* identified five family subtypes for *C. hominis* {IaA20 (n=6), IaA26 (n=1); lbA10G2 (n=1), IdA21 (n=1) IeA11G3T3 (n=1)}, and six family subtypes for *C. parvum* {IIaA17G1R1 (n=6), IIaA19G2R1 (n=3), IIaA15G2R1(n=3) IIaA16G1R1 (n=1), IIaA17G2R1(n=1), IIaA20G1R1(n=1)}. Subtyping analysis of the four *Cryptosporidium* positive samples obtained from *Colobus guereza* revealed the subtype ‘IaA20’. ( see line 148-153 page 8 )

*”*
2. Like for comment 1, descriptions of results and discussion of the data do not delineate between rural and urban populations in a clear manner. The manuscript would be improved by more clearly describing the data from the two sampling locations.

**Response**: - We have presented and discussed the data stratifying it by the urban/rural regions where the sample was collected. “

Added text in the results and discussion

Result

A significantly greater proportion of samples collected from monkey in urban (i.e. Hawassa, 42.5% ) area was positive for *Cryptosporidium* compared to samples obtained from monkey in the rural area (i.e., Wurgissa , 23.44%) (*P=* 0.017) (see page 8-9 line 127-130)

A total of 12 subtypes { IIaA17G1R1(6), IIaA19G2R1(3), IIaA15G2R1(2), IIaA16G2R1(1) IIaA16G1R1(1) IIaA17G2R1(1), IIaA20G1R1(1), IaA20 (5), IdA21(1) lbA10G2(1), IeA11G3T3(1), IaA26(1) )} were seen from NHPs located in Rural Wurgissa but only two subtypes { IaA20(2), IIaA15G2R1 (1)}were identified from monkeys in the Hawassa town ( Table 2 ). (” See page 9 line 145 -149)

**Discussion**

“A higher prevalence of *Cryptosporidium* infection was seen among NHPs living in the urban area compared to the NHPs living in the rural village. This difference might have resulted from ecologic factors (e.g., fecal contaminated water and close habitations), which may facilitate transmission of the parasite between NHPs and humans.” (See line 206-209, page 21).

3. Results: when describing prevalence results, you describe a significant difference between males and females but don’t give the prevalence in each of these groups. Update text to include these values.

**Response**: We have provided prevalence data for males and females in the revised submission. It reads “The prevalence of *Cryptosporidium* infection was higher in female (35.4%) than male (14.70%) monkeys and the difference was statistically significant (p=0.002) (see line 124-126, page 11.”

4. The tree does not seem to be necessary? It is not contributing to the results or discussion of the manuscript as data from the tree are not described or used to explain any aspects of this study.

**Response**: - We acknowledge the reviewer’s comment and removed the phylogenetic tree from the revised manuscript.
5. What is the purpose of the last sentence of the results section? Those subtypes are described above. Why are they listed as additional here? I think this could be resolved by better describing data from each species and study site as suggested in comments 1 and 2.

**Response** : - We have revised the text in the last paragraph of the result section. We have presented data stratifying by monkey spp. and region as described above.

The revised text in the last paragraph reads “ “ Subtyping analysis of the *C. parvum* and *C. hominis* isolates from *Chlorocebus aethiops* identified five family subtypes for *C. hominis* {IaA20 (n=6), IaA26 (n=1); lbA10G2 (n=1), IdA21 (n=1) IeA11G3T3 (n=1)}, and six family subtypes for *C. parvum* {IIaA17G1R1 (n=6), IIaA19G2R1 (n=3), IIaA15G2R1(n=3) IIaA16G1R1 (n=1), IIaA17G2R1(n=1), IIaA20G1R1(n=1)}. Only one family subtype (IaA20) was identified from *Colobus guereza*. A total of 12 subtypes {IIaA17G1R1(6), IIaA19G2R1(3), IIaA15G2R1(2), IIaA16G2R1(1) IIaA16G1R1(1) IIaA17G2R1(1), IIaA20G1R1(1), IaA20 (5), IdA21(1) lbA10G2(1), IeA11G3T3(1), IaA26(1) )} were seen from NHPs located in Rural Wurgissa but only two subtypes { IaA20(2), IIaA15G2R1 (1)}were identified from monkeys in the Hawassa town ( Table 2 ) (line 148-157, page8 ).

6. In the discussion the language that parvum and hominis have broadened their hosts from humans to NHPs should be revised. As you point out the presence of these organisms in NHPs is not well surveyed at the molecular level, and you cannot distinguish between shifts in host range versus observations in new hosts based on this study alone.

**Response**: - We thank the reviewer for the suggestion. We have revised the text. It reads as “These reports suggest that *C. hominis*, which was initially described as infectious to humans, may potentially expand its hosts from humans to NHPs. (lines 181, 182 ; page 10)

6. PLOS authors have the option to publish the peer review history of their article ([what does this mean?](https://journals.plos.org/plosone/s/editorial-and-peer-review-process#loc-peer-review-history)). If published, this will include your full peer review and any attached files.
If you choose “no”, your identity will remain anonymous but your review may still be made public.

Do you want your identity to be public for this peer review? For information about this choice, including consent withdrawal, please see our [Privacy Policy](https://www.plos.org/privacy-policy).

Reviewer #1: No

[NOTE: If reviewer comments were submitted as an attachment file, they will be attached to this email and accessible via the submission site. Please log into your account, locate the manuscript record, and check for the action link "View Attachments". If this link does not appear, there are no attachment files.]

While revising your submission, please upload your figure files to the Preflight Analysis and Conversion Engine (PACE) digital diagnostic tool, <https://pacev2.apexcovantage.com/>. PACE helps ensure that figures meet PLOS requirements. To use PACE, you must first register as a user. Registration is free. Then, login and navigate to the UPLOAD tab, where you will find detailed instructions on how to use the tool. If you encounter any issues or have any questions when using PACE, please email PLOS at [figures@plos.org](mailto:figures@plos.org). Please note that Supporting Information files do not need this step.

*In compliance with data protection regulations, you may request that we remove your personal registration details at any time.* [*(Remove my information/details)*](https://www.editorialmanager.com/pone/login.asp?a=r)*. Please contact the publication office if you have any questions.*
